# Imaging 6-Phosphogluconolactonase Activity in Brain Tumors *In Vivo* Using Hyperpolarized δ-[1-^13^C]gluconolactone

**DOI:** 10.3389/fonc.2021.589570

**Published:** 2021-04-15

**Authors:** Georgios Batsios, Céline Taglang, Peng Cao, Anne Marie Gillespie, Chloé Najac, Elavarasan Subramani, David M. Wilson, Robert R. Flavell, Peder E. Z. Larson, Sabrina M. Ronen, Pavithra Viswanath

**Affiliations:** Department of Radiology and Biomedical Imaging, University of California, San Francisco, CA, United States

**Keywords:** magnetic resonance spectroscopy/imaging (MRS/I), hyperpolarized ^13^C MRS, dynamic nuclear polarization (DNP), pentose phosphate pathway (PPP), glioblastoma, brain tumors, 6-phosphogluconolactonase (PGLS), metabolic therapy

## Abstract

**Introduction:**

The pentose phosphate pathway (PPP) is essential for NADPH generation and redox homeostasis in cancer, including glioblastomas. However, the precise contribution to redox and tumor proliferation of the second PPP enzyme 6-phosphogluconolactonase (PGLS), which converts 6-phospho-δ-gluconolactone to 6-phosphogluconate (6PG), remains unclear. Furthermore, non-invasive methods of assessing PGLS activity are lacking. The goal of this study was to examine the role of PGLS in glioblastomas and assess the utility of probing PGLS activity using hyperpolarized δ-[1-^13^C]gluconolactone for non-invasive imaging.

**Methods:**

To interrogate the function of PGLS in redox, PGLS expression was silenced in U87, U251 and GS2 glioblastoma cells by RNA interference and levels of NADPH and reduced glutathione (GSH) measured. Clonogenicity assays were used to assess the effect of PGLS silencing on glioblastoma proliferation. Hyperpolarized δ-[1-^13^C]gluconolactone metabolism to 6PG was assessed in live cells treated with the chemotherapeutic agent temozolomide (TMZ) or with vehicle control. ^13^C 2D echo-planar spectroscopic imaging (EPSI) studies of hyperpolarized δ-[1-^13^C]gluconolactone metabolism were performed on rats bearing orthotopic glioblastoma tumors or tumor-free controls on a 3T spectrometer. Longitudinal 2D EPSI studies of hyperpolarized δ-[1-^13^C]gluconolactone metabolism and T2-weighted magnetic resonance imaging (MRI) were performed in rats bearing orthotopic U251 tumors following treatment with TMZ to examine the ability of hyperpolarized δ-[1-^13^C]gluconolactone to report on treatment response.

**Results:**

PGLS knockdown downregulated NADPH and GSH, elevated oxidative stress and inhibited clonogenicity in all models. Conversely, PGLS expression and activity and steady-state NADPH and GSH were higher in tumor tissues from rats bearing orthotopic glioblastoma xenografts relative to contralateral brain and tumor-free brain. Importantly, [1-^13^C]6PG production from hyperpolarized δ-[1-^13^C]gluconolactone was observed in live glioblastoma cells and was significantly reduced by treatment with TMZ. Furthermore, hyperpolarized δ-[1-^13^C]gluconolactone metabolism to [1-^13^C]6PG could differentiate tumor from contralateral normal brain *in vivo*. Notably, TMZ significantly reduced 6PG production from hyperpolarized δ-[1-^13^C]gluconolactone at an early timepoint prior to volumetric alterations as assessed by anatomical imaging.

**Conclusions:**

Collectively, we have, for the first time, identified a role for PGLS activity in glioblastoma proliferation and validated the utility of probing PGLS activity using hyperpolarized δ-[1-^13^C]gluconolactone for non-invasive *in vivo* imaging of glioblastomas and their response to therapy.

## Introduction

Gliomas are the most common form of adult-onset primary malignant brain tumors with 25,130 cases expected to be diagnosed in 2021 (1). Gliomas are traditionally classified by histology, in increasing order of malignancy from grade I to grade IV. In 2016, the World Health Organization revised the criteria for glioma classification to include molecular characteristics (2, 3). High-grade (grade IV) tumors that are isocitrate dehydrogenase wild-type are now considered primary glioblastomas. They account for approximately half of all glioma cases and have a very dismal prognosis with a 5-year patient survival rate of <7% (1). The current standard of care for glioblastomas consists of maximal safe surgical resection combined with radiotherapy and chemotherapy with temozolomide (TMZ) (4, 5). Nevertheless, overall patient survival remains short at ~14.6 months (4, 5). In order to improve patient outcomes, there is a need to identify biological processes that are critical for tumor proliferation as well as non-invasive imaging methods that can inform on tumor biology and response to therapy (6).

**Figure 1:**
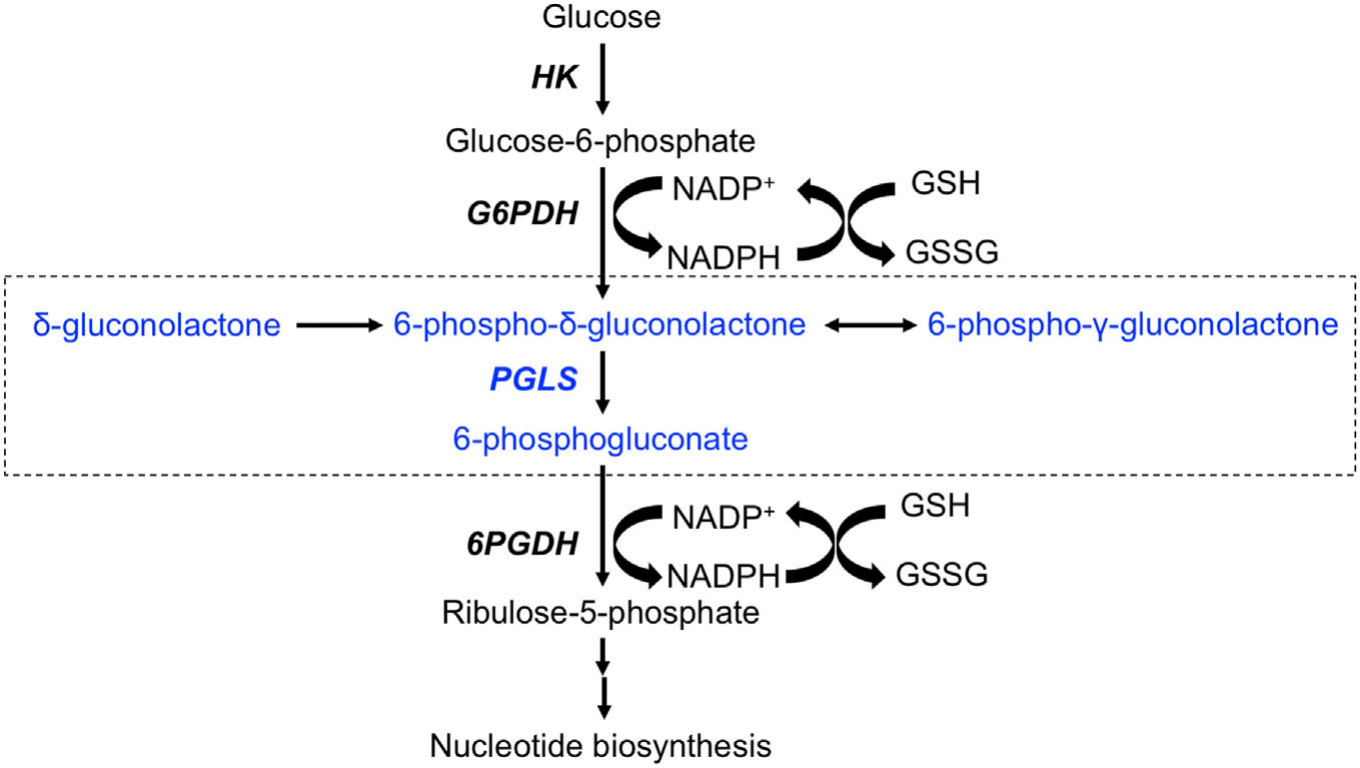
Schematic illustration of the PPP. The portion of the pathway illustrating entry of δ-[1-^13^C]gluconolactone and metabolism to [1-^13^C]6PG are highlighted in blue and enclosed in a box. HK, Hexokinase; G6PDH, glucose-6-phospate dehydrogenase; PGLS, 6-phosphogluconolactonase; 6PGDH, 6-phosphogluconate deydrogenase.

Metabolic reprogramming has emerged as a fundamental hallmark of cancer (7–9). Nutrient uptake and metabolism are tightly regulated in normal somatic cells. All tumor cells, including glioblastomas, acquire oncogenic mutations that facilitate rewiring of metabolic pathways to allow for the increased demand for biosynthetic intermediates and redox metabolites needed to support uncontrolled proliferation (8, 9). The best studied metabolic hallmark in cancer is the Warburg effect wherein tumor cells increase glucose uptake and concomitant lactate production even under aerobic conditions (10). Although less is known about glucose metabolism through the pentose phosphate pathway (PPP), emerging evidence points to upregulation of PPP flux in tumor tissues, including in glioblastomas (11–13). The PPP branches from glycolysis at the level of glucose-6-phosphate, which is oxidized by glucose-6-phosphate dehydrogenase to 6-phospho-δ-gluconolactone while reducing NADP^+^ to NADPH (see **Figure 1**). 6-phospho-δ-gluconolactone, which is in equilibrium with 6-phospho-γ-gluconolactone (14, 15), is then hydrolyzed by 6-phosphogluconolactonase (PGLS) to generate 6-phosphogluconate (6PG). Subsequent formation of ribulose-5-phosphate occurs *via* oxidative decarboxylation of 6PG by 6-phosphogluconate dehydrogenase with the concomitant generation of NADPH. Several studies have identified essential roles for glucose-6-phosphate dehydrogenase and 6-phosphogluconate dehydrogenase in the generation of precursors for ribonucleotide synthesis, and thereby, nucleic acid synthesis (12, 16, 17). In addition, these enzymes have been identified as the major sources of NADPH, which, in turns, plays an essential role in maintaining glutathione (GSH) in the reduced state and thereby, combating oxidative stress (11, 12, 16, 18). Glucose-6-phosphate dehydrogenase and 6-phosphogluconate dehydrogenase are, therefore, therapeutic targets in cancer (16, 19, 20). However, to date, studies examining the contribution of PGLS to redox maintenance or tumor proliferation in cancer are lacking.

Magnetic resonance spectroscopy (MRS) is a method of interrogating metabolism in a non-invasive manner in cells, animals and human patients (21–23). By tracking metabolites and metabolic fluxes that differ between tumor and normal tissue, MRS has the ability to monitor tumor burden and response to therapy. ^1^H MRS monitors steady-state metabolite levels while ^13^C MRS monitors metabolic fluxes. However, thermally polarized ^13^C MRS is limited in sensitivity, which hampers its clinical utility. In this context, the recent introduction of hyperpolarization *via* dissolution dynamic nuclear polarization allows >10,000-fold enhancement in the signal to noise ratio (SNR) of the ^13^C signal relative to the thermally polarized agent. This has allowed for rapid, non-invasive, pathway-specific, real-time monitoring of several metabolic and physiological processes (23–26). The most successful hyperpolarized ^13^C agent is hyperpolarized [1-^13^C]pyruvate, which monitors the Warburg effect-mediated flux from pyruvate to lactate and has been used to monitor tumor burden and response to therapy in several cancers, including glioblastomas (23–25). Interestingly, a recent study demonstrated hyperpolarization of δ-[1-^13^C]gluconolactone and metabolism to 6PG *ex vivo* in isolated perfused liver (27). δ-[1-^13^C]gluconolactone enters the cell *via* glucose transporters and is trapped by phosphorylation to 6-phospho-δ-[1-^13^C]gluconolactone (28, 29), which is then metabolized by PGLS to [1-^13^C]6PG. Hyperpolarized δ-[1-^13^C]gluconolactone, therefore, has the potential to monitor PGLS activity *in vivo*.

The goal of our study was to examine the role of PGLS in glioblastoma redox and proliferation and evaluate whether using hyperpolarized δ-[1-^13^C]gluconolactone to probe PGLS activity allows glioblastoma imaging and assessment of response to therapy *in vivo*. Our findings indicate that PGLS contributes to redox maintenance and is linked to proliferation in multiple glioblastoma models, including a clinically relevant patient-derived model. Importantly, hyperpolarized δ-[1-^13^C]gluconolactone metabolism to [1-^13^C]6PG serves to differentiate tumor from normal brain and to report on response to TMZ in rats bearing orthotopic tumor xenografts *in vivo*.

## Methods

### δ-[1-^13^C]gluconolactone Probe Synthesis and Hyperpolarization

δ-[1-^13^C]gluconolactone was synthesized and polarized as previously described (27, 30). After synthesis a small amount of the powder was dissolved in deuterated dimethyl sulfoxide (DMSO) and MR spectra were acquired using a 9.4 T Bruker spectrometer to confirm δ-[1-^13^C]gluconolactone synthesis and absence of any contaminant. 2 M δ-[1-^13^C]gluconolactone was dissolved in 3:1 water: glycerol and mixed with 15 mM trityl radical OX063 and polarized in a HyperSense polarizer (3.35 T, 1.4 K, Oxford Instruments, UK) for ~1.5 h. After maximal polarization was achieved, the sample was dissolved in phosphate-buffered saline (pH~7) to a final concentration of 8 mM for cell studies and 37.8 mM for *in vivo* studies.

### Relaxation and Polarization Levels

Following dissolution, 2 ml of hyperpolarized δ-[1-^13^C]gluconolactone was rapidly transferred to a horizontal 3 T scanner (BioSpec 105 mm bore diameter, Bruker) to evaluate T_1_ (n = 3, TR = 3 s/FA = 10°) or to a vertical 11.7 T MR system (INOVA, Agilent Technologies) to evaluate percent polarization (n = 3, TR = 300 s/FA = 90°/NA = 16) and T_1_ (n = 3, TR = 3 s/FA = 13°). Spectra were processed by peak integration using MestreNova (v12.0.4, Mestrelab, Spain). For determination of T_1_, the quantified peak integrals were corrected for flip angle and fitted with a mono-exponential curve. The liquid-state polarization and SNR improvement were evaluated by comparing the first hyperpolarized spectrum of the dynamic set to the corresponding thermal equilibrium spectrum after correction for flip angle, number of averages and back calculating the value to the time of dissolution (18 to 25 s prior to first spectra acqusition).

### Cell Culture

The U87 and U251 models are standard glioblastoma models that were obtained from ATCC. The GS2 model is a patient-derived glioblastoma model that was obtained from the UCSF Brain Tumor Center Preclinical Therapeutics Core. All cell lines were maintained in Dulbecco’s modified Eagle’s medium (DMEM) supplemented with 10% fetal calf serum, 2 mM glutamine, and 100 U/ml each of penicillin and streptomycin under normoxic conditions for no more than 30 passages before use. Cells were authenticated by short tandem repeat fingerprinting (Cell Line Genetics) within 6 months of any study. TMZ (Sigma-Aldrich) was dissolved in DMSO and added to cells at a final concentration of 100 μM as previously described (31). DMSO was used as vehicle control.

### PGLS Expression and Activity

PGLS expression in tissue samples was assessed by quantitative RT-PCR using a SYBR Green assay kit with the following primers: forward primer (TGTGGCAACTGGAGAAGGCAAG), reverse primer (CTCGTCCAAGAACCAGCACAGT). PGLS expression was silenced by RNA interference using the SMARTpool siGENOME human PGLS siRNA (set of 4 siRNAs: GAUUGUGGCUCCCAUCAGU, CACACUACCUGUCCUGAAU; GCAAGGCAGCUGUUCUGAA; CGGCUGAGGACUACGCCAA; Dharmacon). siGENOME Non-Targeting siRNA Pool #1 (set of 4: UAGCGACUAAACACAUCAA, UAAGGCUAUGAAGAGAUAC, AUGUAUUGGCCUGUAUUAG, AUGAACGUGAAUUGCUCAA; Dharmacon) was used as control. PGLS activity was assayed spectrophotometrically in a coupled enzyme assay by measuring the reduction of NADP^+^ to NADPH during breakdown of 6PG produced by PGLS from δ-gluconolactone in presence of 6-phosphogluconate dehydrogenase as previously described (32). Briefly, assays were started by addition of 0.2 mM δ-gluconolactone and 0.6 mM NADP^+^ to 50 mM Tris-HCl buffer, pH 7 containing 2 mM MgCl_2_ and 5 mM ATP. The absorbance at 340 nm was monitored following addition of 0.2 U/ml of 6-phosphogluconate dehydrogenase and cell or tumor extract (50 μg).

### Redox Measurements

Steady-state levels of NADP^+^, NADPH, GSH, oxidized glutathione (GSSG) and reactive oxygen species (ROS) were measured by spectrophotometry or fluorimetry using commercially available kits (Abcam). All measurements were done in triplicates (n = 3).

### Hyperpolarized ^13^C MRS Studies in Live Cells

Hyperpolarized δ-[1-^13^C]gluconolactone was polarized as described above. An aliquot corresponding to a final concentration of 8 mM was polarized for ~1.5 h, dissolved in isotonic buffer (phosphate-buffered saline, pH 7.5) and added to an NMR tube containing a suspension of ~3 x 10^7^ live cells (33). ^13^C spectra were acquired on a Varian 11.7 T spectrometer with a 13° flip angle and 3 s TR for 300 s. Data analysis was performed using MestreNova and the area under the curve (AUC) for [1-^13^C]6PG was normalized to the AUC for substrate (δ-[1-^13^C]gluconolactone) and to cell number. Previous studies indicate that δ-gluconolactone is in equilibrium with γ-gluconolactone in aqueous solution (14, 27). In order to confirm that the differences in 6PG production in our studies were not the result of differences in the relative levels of δ- and γ-gluconolactone, we also evaluated the ratio of [1-^13^C]6PG to the combined signal from hyperpolarized δ-[1-^13^C]gluconolactone and γ-[1-^13^C]gluconolactone (henceforth referred to as total [1-^13^C]gluconolactone) and to cell number.

### *In Vivo* MRI and Hyperpolarized ^13^C MRS Studies

#### Orthotopic Tumor Generation and Treatment

All studies were performed under the UCSF Institutional Animal Care and Use Committee approval (IACUC Protocol No: AN170079). Orthotopic tumors were generated by intracranial implantation of ~3 x 10^5^ cells per rat into athymic male nu/nu rats (5 weeks old) as previously described (33, 34). Tumor volume was monitored by T2-weighted MRI as described below and hyperpolarized δ-[1-^13^C]gluconolactone metabolism was assessed once tumors reached a volume of 0.28 ± 0.08 cm^3^. For assessment of treatment response in rats bearing orthotopic U251 tumors, TMZ was administered intraperitoneally at a dose of 50 mg/kg in 20% DMSO in saline when tumors reached a volume of 0.27 ± 0.05 cm^3^. The animals were treated with TMZ daily for a week and subsequently treatment was shortened to twice a week. A total of 16 rats were investigated. 6 rats were implanted with U87 tumors and 4 rats with U251 tumors. 6 rats were used as age-matched tumor-free controls.

#### Data Acquisition

All measurements were performed on a horizontal 3 T scanner (BioSpec 105 mm bore diameter, Bruker). U87-bearing and tumor-free control animals were imaged using a dual-tuned ^1^H-^13^C linear-linear volume coil (40 mm inner diameter), while U251-bearing rats were imaged using a ^1^H quadrature volume coil (72mm inner diameter) and a ^13^C quadrature volume coil (72mm inner diameter). Animals were anesthetized and maintained using isoflurane (1 - 2% in O_2_) and placed headfirst in the prone position with a respiratory sensor. Axial and sagittal anatomical T2-weighted images were recorded using a spin echo (TurboRARE) sequence (TE/TR = 64/3484 ms, FOV = 35 x 35 mm^2^, 256 x 256, slice thickness = 1 mm, NA = 5) and used to evaluate tumor location and size. Hyperpolarized ^13^C MRS studies were performed following injection of 2.2 ml of hyperpolarized δ-[1-^13^C]gluconolactone (prepared as described above) *via* a tail-vein catheter over 15 s. Spectroscopic imaging was performed using a dynamic 2D flyback spectral spatial echo planar spectroscopic imaging (EPSI) sequence with flip angles of 15.3° on [1-^13^C]6PG, 3.4° on δ-[1-^13^C]gluconolactone and 12° on γ-[1-^13^C]gluconolactone. The spatial resolution of the ^13^C MRSI was 4.375 x 4.375 x 8 mm^3^ (for U87-bearing and tumor-free control rats) and 5.375 x 5.375 x 8 mm^3^ (for U251-bearing rats), while the temporal resolution was 3 s. The spectral resolution was 128 points over 20 ppm. Data acquisition was initiated with the start of injection of hyperpolarized δ-[1-^13^C]gluconolactone.

#### Data Analysis

Tumor volume was evaluated as the sum of manually contoured tumor areas in each slice multiplied by slice thickness using an in-house IDL-based software (33–35). 2D EPSI data were processed using in-house Matlab codes (R2019a, Mathworks) that have previously been described (33, 34) and are available from a github repository (https://github.com/ViswanathLab/EPSI). For each voxel at every time point, spectra were analyzed after a 5 Hz line broadening by determining the area under each peak by integration. For generation of metabolic heatmaps, raw data were interpolated from an 8 x 8 matrix to a 256 x 256 matrix using the *imresize* function of Matlab and the Lanczos-2 interpolation algorithm and normalizing to noise, which was evaluated as the standard deviation of the real part of the signal in a voxel outside of the brain. These maps were used to generate the ratio of product ([1-^13^C]6PG) to substrate (δ-[1-^13^C]gluconolactone or total [1-^13^C]gluconolactone). Additionally, the SNR of each metabolite ([1-^13^C]6PG, δ-[1-^13^C]gluconolactone and γ-[1-^13^C]gluconolactone) and the ratio of [1-^13^C]6PG to δ-[1-^13^C]gluconolactone or to total [1-^13^C]gluconolactone was assessed in a 28.13 mm^3^ volume from tumor, normal-appearing contralateral appearing brain or healthy brain regions.

### Statistical Analysis

All results are expressed as mean ± standard deviation. Uppaired two-tailed student’s t-test with unequal variance was used to assess the statistical significance of differences in hyperpolarized ^13^C MRS data, PGLS expression and activity, NADP^+^, NADPH, GSH, GSSG and ROS between tumor-bearing animals and controls and in cell experiments (p<0.05 considered significant).

## Results

### PGLS Is Important for Redox Homeostasis and Proliferation in Glioblastoma Cells

**Figure 2:**
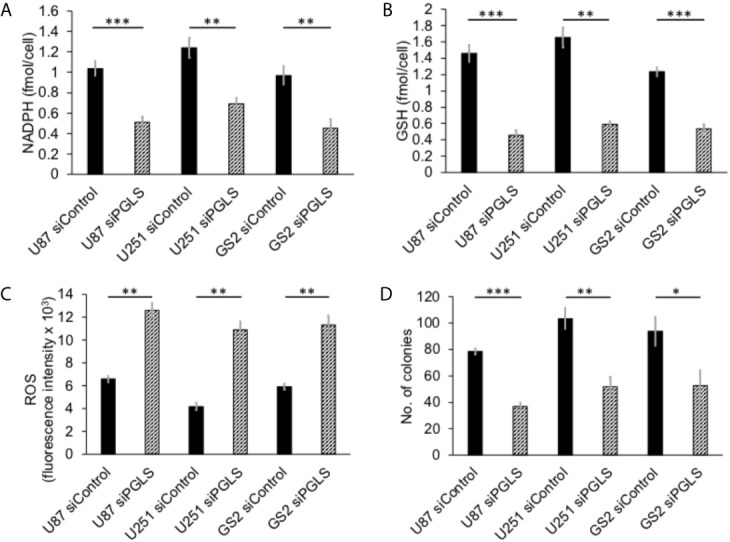
PGLS is linked to redox in glioblastoma cells. Effect of PGLS silencing by RNA interference on NADPH **(A)**, GSH **(B)**, ROS **(C)** and clonogenicity **(D)** in the U87, U251 and GS2 glioblastoma models (n = 3). * indicates p < 0.05, ** indicates p < 0.01, *** indicates p < 0.001.

PGLS functions in the PPP to generate 6PG, which is decarboxylated by 6-phosphogluconate dehydrogenase with the concomitant generation of NADPH (refer to **Figure 1**) (11, 14). NADPH, in turn, maintains GSH in the reduced state (11, 36). In order to determine whether PGLS plays a role in the generation of NADPH and the maintenance of GSH levels, we examined the effect of silencing PGLS in U87, U251 and GS2 glioblastoma cells on steady-state levels of NADPH and GSH. PGLS silencing (see **Supplementary Figures 1A, B** for verification of PGLS knockdown) significantly reduced steady-state levels of NADPH (**Figure 2A**) and GSH (**Figure 2B**) in the U87, U251 and GS2 models. There was no change in levels of NADP^+^ (**Supplementary Figure 1C**) or GSSG (**Supplementary Figure 1D**) following PGLS silencing. Importantly, PGLS silencing significantly increased ROS levels (**Figure 2C**), an effect that was associated with significantly reduced clonogenicity (**Figure 2D**) in all 3 models. Collectively, these results indicate that PGLS plays an important role in glioblastoma proliferation and contributes to redox homeostasis *via* generation of NADPH and GSH and concomitant reduction of oxidative stress.

### PGLS Expression and Activity Are Upregulated in Tumor Tissue Relative to Normal Brain *In Vivo*

Next, we examined expression and activity of PGLS in tumor tissue and normal-appearing contralateral brain from rats bearing orthotopic U87, U251 or GS2 tumors. We also examined healthy brain from tumor-free control rats as additional controls. As shown in **Supplementary Figures 2A–F**, PGLS expression and activity were significantly higher in tumor tissue relative to both normal-appearing contralateral brain and healthy tumor-free normal brain tissue. There was no significant difference in PGLS expression or activity between normal-appearing contralateral brain and healthy tumor-free brain (**Supplementary Figures 2A–F**). Concomitantly, levels of NADPH (**Supplementary Figures 3A–C**) and GSH (**Supplementary Figures 4A–C**) were significantly higher while ROS levels (**Supplementary Figures 5A–C**) were significantly lower in tumor tissues relative to normal-appearing contralateral brain and healthy brain from tumor-free rats for all 3 models. In line with PGLS expression and activity, there was no significant difference in NADPH (**Supplementary Figures 3A-C**), GSH (**Supplementary Figures 4A-C**) or ROS (**Supplementary Figures 5A-C**) between normal-appearing contralateral brain and healthy tumor-free brain. There was also no difference in NADP^+^ (**Supplementary Figures 6A-C**) or GSSG (**Supplementary Figures 7A-C**) between tumor, normal-appearing contralateral brain and tumor-free healthy brain in any of the models. Taken together with the data from our cell studies, these results suggest that PGLS is potentially linked to redox homeostasis *in vivo* and provide a rational basis for monitoring PGLS activity *via* hyperpolarized δ-[1-^13^C]gluconolactone in glioblastomas *in vivo*.

### Metabolism of Hyperpolarized δ-[1-^13^C]gluconolactone to [1-^13^C]6PG Can Be Observed in Live Glioblastoma Cells and Is Reduced in Response to Therapy

**Figure 3:**
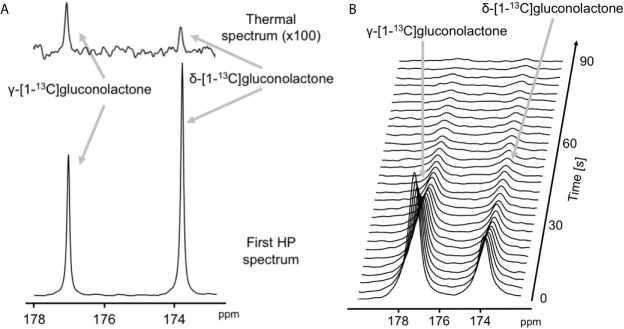
Characterization of hyperpolarized δ-[1-^13^C]gluconolactone. **(A)** Representative thermal equilibrium (top) and hyperpolarized ^13^C MR spectrum (bottom) of δ-[1-^13^C]gluconolactone. **(B)** Representative stack plot of ^13^C MR spectra of hyperpolarized δ-[1-^13^C]gluconolactone in solution (temporal resolution 3 s).

**Figure 4:**
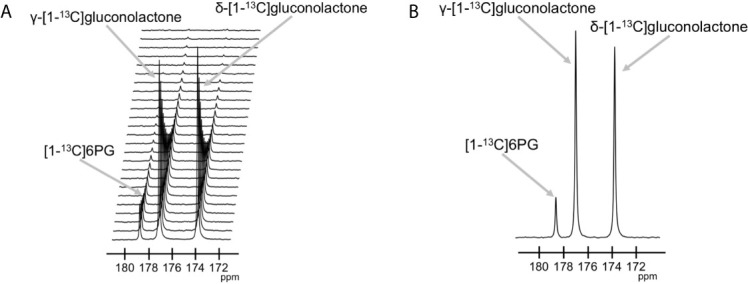
Hyperpolarized δ-[1-^13^C]gluconolactone metabolism to [1-^13^C]6PG can be observed in live glioblastoma cells. **(A)** Representative ^13^C MR spectral array showing dynamic production of [1-^13^C]6PG from hyperpolarized δ-[1-^13^C]gluconolactone in live U87 cells. Peaks for δ-[1-^13^C]gluconolactone, γ-[1-^13^C]gluconolactone and [1-^13^C]6PG can be observed. **(B)** Summed ^13^C MR spectrum showing [1-^13^C]6PG production from hyperpolarized δ-[1-^13^C]gluconolactone in live U87 cells.

The feasibility of non-invasively assessing 6PG production from hyperpolarized δ-[1-^13^C]gluconolactone has previously been shown in normal isolated perfused liver at 9.4 T (27). Here, we examined whether hyperpolarized δ-[1-^13^C]gluconolactone metabolism to [1-^13^C]6PG can be observed in glioblastoma models. To this end, we first measured T_1_ and polarization values for hyperpolarized δ-[1-^13^C]gluconolactone. As shown in **Figures 3A, B**, we were able to detect δ-[1-^13^C]gluconolactone (173.8 ppm) with a calculated T_1_ of 31.7 ± 4.7 s (n = 3) at 3 T and 15.7 ± 0.1 s (n = 3) at 11.7 T. These T_1_ values are consistent with previously published results (17.8 s at 9.4 T) once the change in field strength is taken into account (37). The % polarization was 14.7 ± 2.6% (back calculated to time of dissolution) (n = 3) and resulted in a 15634 ± 2600-fold enhancement in SNR. In aqueous solutions, δ-[1-^13^C]gluconolactone is in equilibrium with γ-[1-^13^C]gluconolactone (177 ppm) and both species were observed in our spectra, consistent with previous studies (14, 27). Importantly, following addition to a suspension of live glioblastoma cells, hyperpolarized δ-[1-^13^C]gluconolactone metabolism to [1-^13^C]6PG (178.6 ppm; **Figures 4A, B**) could clearly be observed.

**Figure 5:**
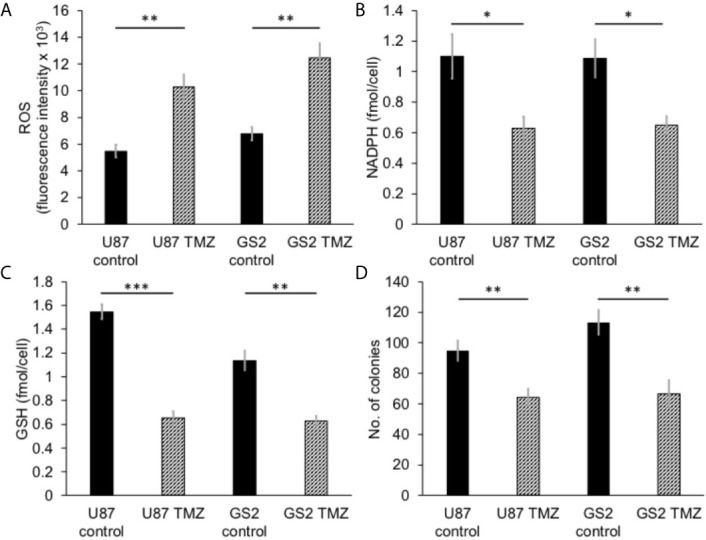
TMZ induces oxidative stress and reduces proliferation in glioblastoma cells. Effect of TMZ on ROS **(A)**, NADPH **(B)**, GSH **(C)** and clonogenicity **(D)** in U87 and GS2 glioblastoma cells (n = 3 each). * indicates p < 0.05, ** indicates p < 0.01, *** indicates p < 0.001.

**Figure 6:**
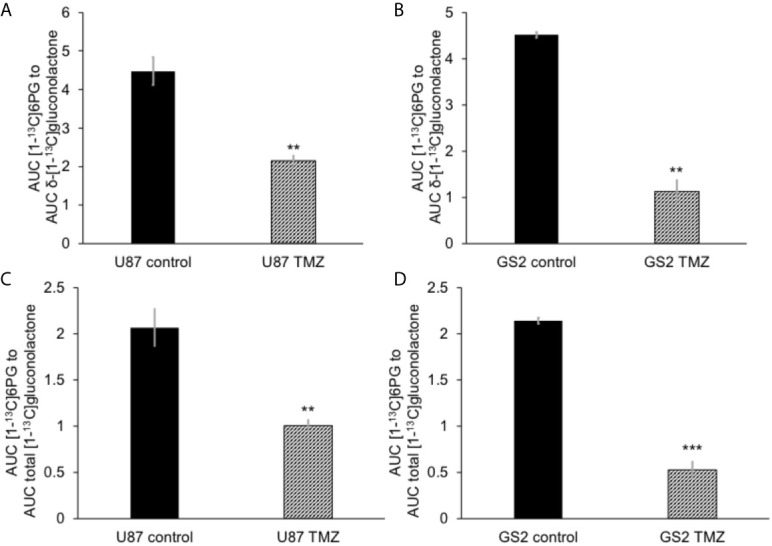
Hyperpolarized δ-[1-^13^C]gluconolactone metabolism to [1-^13^C]6PG is reduced following TMZ treatment in glioblastoma cells. The ratio of [1-^13^C]6PG to δ-[1-^13^C]gluconolactone in TMZ-treated U87 **(A)** and GS2 **(B)** cells. Effect of TMZ on the ratio of [1-^13^C]6PG to total [1-^13^C]gluconolactone in the U87 **(C)** and GS2 **(D)** models (n = 3 each). ** indicates p < 0.01, *** indicates p < 0.001.

We then examined the ability of hyperpolarized δ-[1-^13^C]gluconolactone to report on response to treatment with TMZ, which is standard of care for glioblastoma patients and has been shown to induce oxidative stress in glioblastomas (38, 39). In line with previous studies, we confirmed that TMZ significantly increased levels of ROS (**Figure 5A**) and reduced steady-state NADPH (**Figure 5B**) and GSH (**Figure 5C**), an effect that was associated with inhibition of clonogenicity (**Figure 5D**) in the U87 and GS2 models. There was no significant change in steady-state levels of NADP^+^ (**Supplementary Figure 8A**) or GSSG (**Supplementary Figure 8B**) in TMZ-treated cells in both U87 and GS2 models. Importantly, the ratio of [1-^13^C]6PG to hyperpolarized δ-[1-^13^C]gluconolactone was significantly reduced in TMZ-treated cells relative to vehicle-treated controls in both U87 and GS2 models (**Figures 6A, B**). In order to confirm that the differences in 6PG production between control and TMZ-treated cells were not the result of differences in the relative levels of δ- and γ-gluconolactone, which are in equilibrium (14, 27), we also evaluated the ratio of [1-^13^C]6PG to total [1-^13^C]gluconolactone. As shown in **Figures 6C, D**, TMZ treatment induced a significant drop in the ratio of [1-^13^C]6PG to total [1-^13^C]gluconolactone in both U87 and GS2 models. Collectively, these results indicate that probing PGLS activity using hyperpolarized δ-[1-^13^C]gluconolactone can be used to assess response to therapy in glioblastoma cells.

### Hyperpolarized δ-[1-^13^C]gluconolactone Metabolism to 6PG Can Discriminate Tumor From Contralateral Normal Brain *In Vivo*

**Figure 7:**
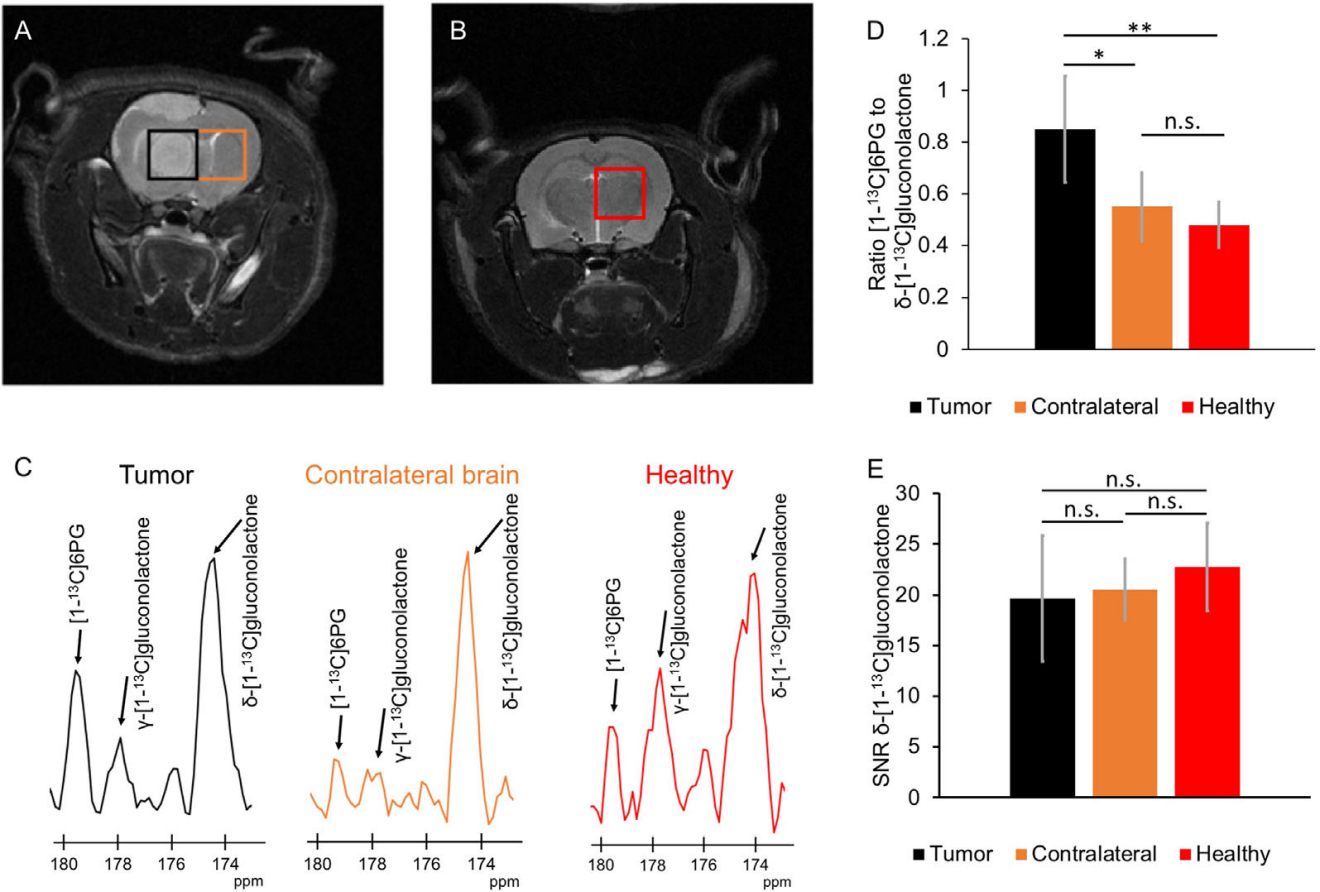
Hyperpolarized δ-[1-^13^C]gluconolactone metabolism to [1-^13^C]6PG can differentiate tumor from normal brain *in vivo*. **(A)** Representative T2-weighted MRI from 2D EPSI studies of hyperpolarized δ-[1-^13^C]gluconolactone metabolism showing placement of the tumor voxel (black) and normal-appearing contralateral brain voxel (orange) in a rat bearing an orthotopic U87 tumor xenograft. **(B)** Representative T2-weighted MRI from 2D EPSI studies in a tumor-free healthy rat. The voxel from healthy brain is shown in red. **(C)** Representative ^13^C MR spectra from tumor (black) and normal-appearing contralateral brain (orange) voxels from a U87 tumor-bearing rat and healthy brain voxel (red) from a tumor-free control rat. Quantification of the ratio of [1-^13^C]6PG to δ-[1-^13^C]gluconolactone **(D)** and the SNR of δ-[1-^13^C]gluconolactone **(E)** in ROIs from tumor (black), normal-appearing contralateral brain (orange) and healthy tumor-free brain (red). n = 6; * indicates p<0.05, ** indicates p<0.01: ns indicates non significance.

Next, we examined whether hyperpolarized δ-[1-^13^C]gluconolactone metabolism to [1-^13^C]6PG can be observed *in vivo.* Following intravenous injection of hyperpolarized δ-[1-^13^C]gluconolactone into rats bearing orthotopic U87 tumor xenografts, the spatial distribution of δ-[1-^13^C]gluconolactone and [1-^13^C]6PG was assessed using a flyback spectral spatial 2D EPSI acquisition scheme (**Figure 7A**). Tumor-free healthy rats were examined as additional controls (**Figure 7B**). As shown in the representative ^13^C spectra in **Figure 7C**, [1-^13^C]6PG was higher in the tumor voxel compared to normal-appearing contralateral brain or to healthy brain from tumor-free rats. Quantification of the SNR of δ-[1-^13^C]gluconolactone, the SNR of [1-^13^C]6PG as well as the ratio of [1-^13^C]6PG to δ-[1-^13^C]gluconolactone or to total [1-^13^C]gluconolactone from a region of interest (ROI) within the tumor, normal-appearing contralateral brain or tumor-free healthy brain confirmed the statistical significance of these findings. The ratio of [1-^13^C]6PG to δ-[1-^13^C]gluconolactone (**Figure 7D**) or to total [1-^13^C]gluconolactone (**Supplementary Figure 9A**) was significantly higher in tumor compared to normal-appearing contralateral brain or healthy brain, while there was no difference between normal-appearing contralateral brain and healthy brain. Similarly, the SNR of [1-^13^C]6PG was significantly higher in tumor relative to normal-appearing contralateral brain and healthy tumor-free brain (**Supplementary Figure 9B**). There was no significant difference in the SNR of the substrate hyperpolarized δ-[1-^13^C]gluconolactone (**Figure 7E**) or total [1-^13^C]gluconolactone (**Supplementary Figure 9C**) between tumor, normal-appearing contralateral brain and tumor-free healthy brain.

**Figure 8:**
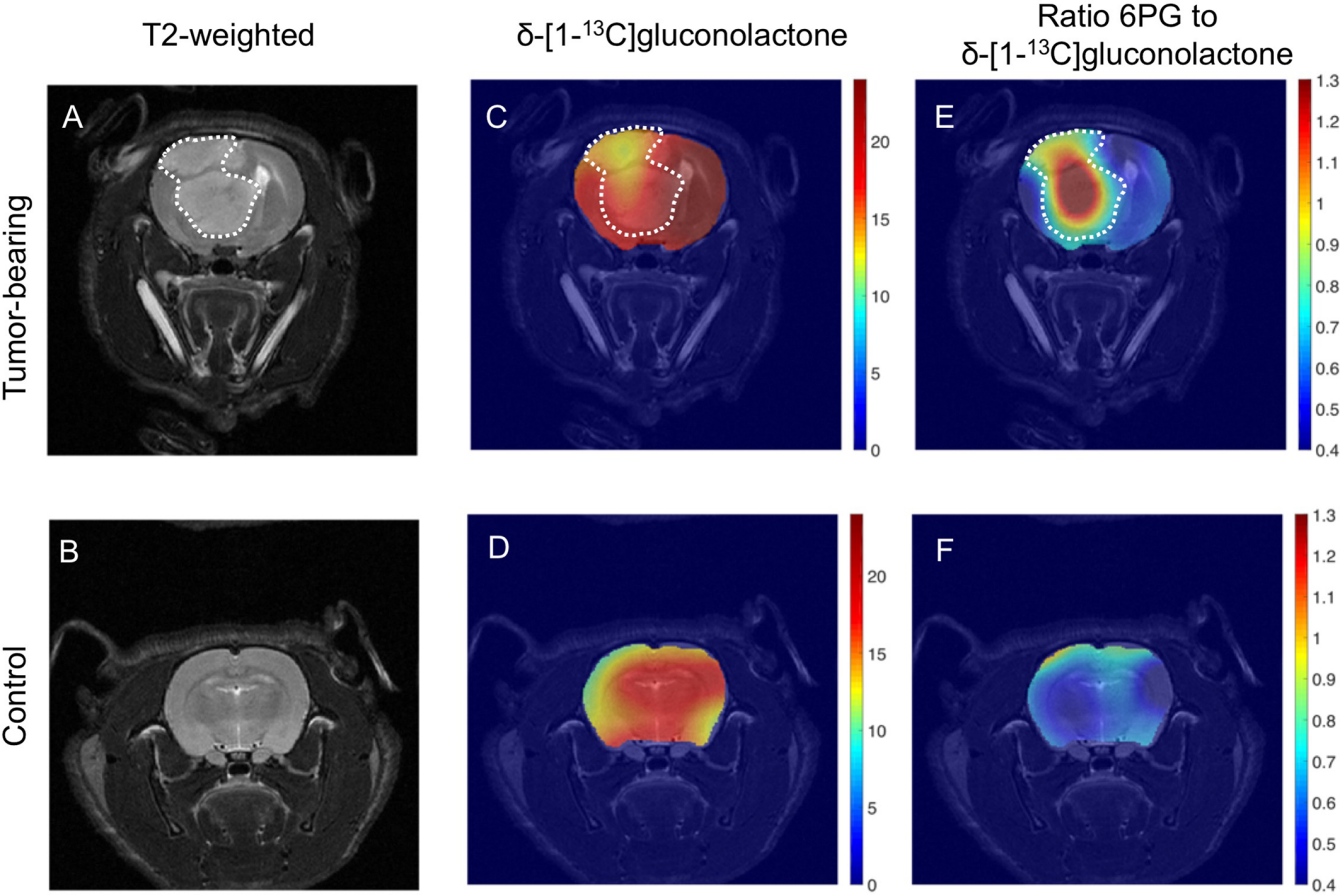
Metabolic imaging of glioblastoma tumors *in vivo* using hyperpolarized δ-[1-^13^C]gluconolactone. Representative T2-weighted MRI **(A, B)** and metabolic heatmaps of the SNR of δ-[1-^13^C]gluconolactone **(C, D)** and the ratio of [1-^13^C]6PG, to δ-[1-^13^C]gluconolactone **(E, F)** from a rat bearing an orthotopic U87 tumor or tumor free healthy control. The tumor region is contoured by white dotted lines.

The ability of hyperpolarized δ-[1-^13^C]gluconolactone metabolism to [1-^13^C]6PG to demarcate tumor from normal brain was further confirmed by examination of metabolic heat maps in which the hyperpolarized ^13^C signal was superimposed over the corresponding anatomical T2-weighted MR images (**Figures 8A, B**). Hyperpolarized δ-[1-^13^C]gluconolactone (**Figures 8C, D**) and total [1-^13^C]gluconolactone (**Supplementary Figures 10C, D**) were evenly distributed throughout the brain of both U87 tumor-bearing and tumor-free control rats. In contrast, metabolic heatmaps of [1-^13^C]6PG (**Supplementary Figures 10A, B**), the ratio of [1-^13^C]6PG to δ-[1-^13^C]gluconolactone (**Figures 8E, F**) and the ratio of [1-^13^C]6PG to total [1-^13^C]gluconolactone (**Supplementary Figures 10E, F**) showed localization of 6PG production to the tumor region relative to normal-appearing contralateral brain and healthy brain from tumor-free rats. Taken together, these results indicate that hyperpolarized δ-[1-^13^C]gluconolactone crosses the blood brain barrier (BBB) and that monitoring [1-^13^C]6PG production from hyperpolarized δ-[1-^13^C]gluconolactone has the potential to non-invasively monitor tumor burden *in vivo*, in line with elevated PGLS expression and activity in glioblastomas relative to normal brain.

### Hyperpolarized δ-[1-^13^C]gluconolactone Can Monitor Response to Therapy *In Vivo*

**Figure 9:**
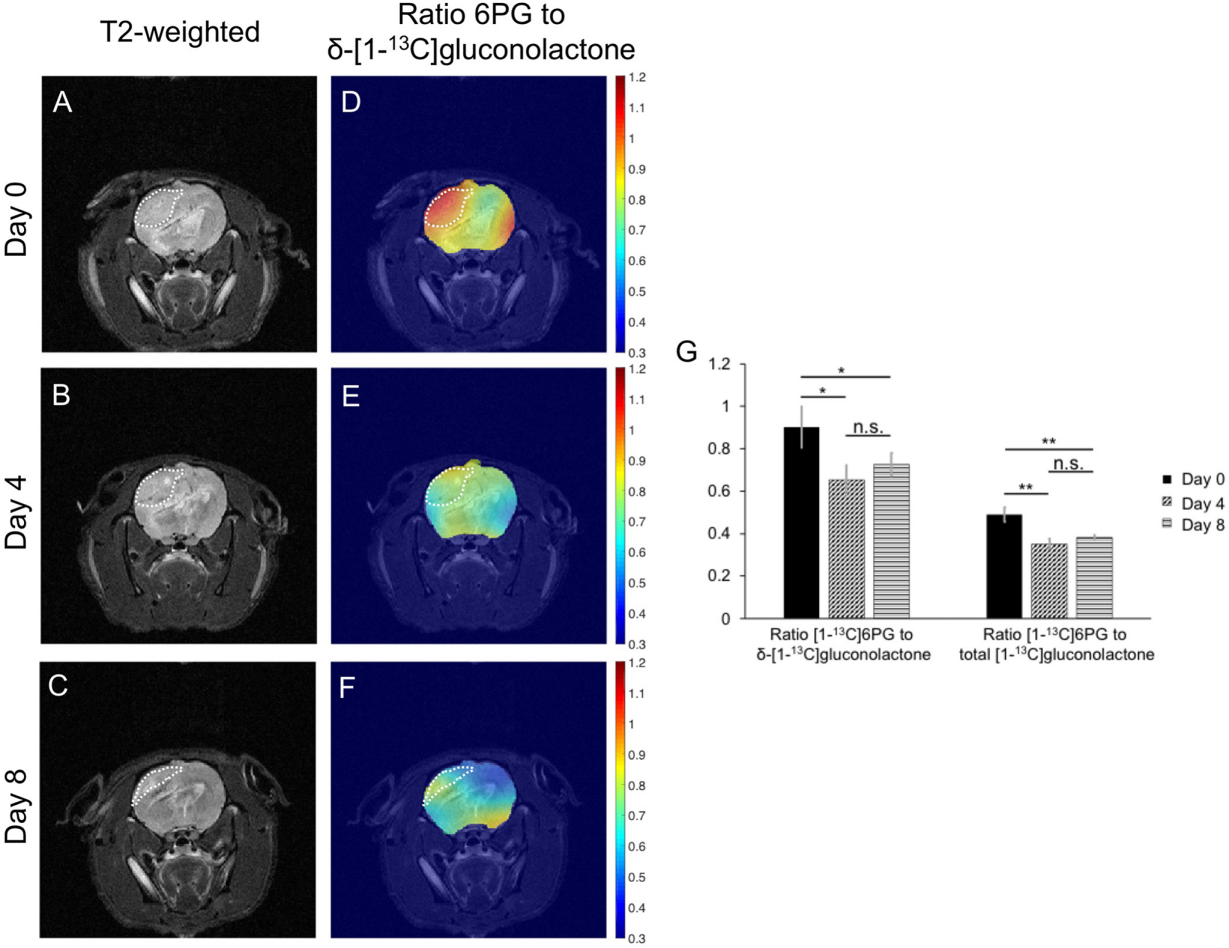
Hyperpolarized δ-[1-^13^C]gluconolactone can be used to monitor response to TMZ *in vivo*. Representative T2-weighted MRI **(A-C)** and metabolic heatmaps of the ratio of [1-^13^C]6PG to δ-[1-^13^C]gluconolactone **(D-F)** from a rat bearing an orthotopic U251 tumor xenograft at day 0, day 4 and day 8 following treatment with TMZ. The tumor region is contoured by white dotted lines. **(G)** Quantification of the ratio of [1-^13^C]6PG to δ-[1-^13^C]gluconolactone or the ratio of [1-^13^C]6PG to total [1-^13^C]gluconolactone from an ROI within the tumor at day 0, day 4 and day 8 of TMZ treatment in rats bearing orthotopic U251 tumor xenografts (n = 4 at day 0 and n = 3 at day 4 and 8). * indicates p<0.05, ** indicates p<0.01: ns indicates non significance.

Finally, based on the results of our cell studies suggesting that hyperpolarized [1-^13^C]6PG production is reduced following TMZ treatment (see **Figures 6A-D**), we examined the ability of hyperpolarized δ-[1-^13^C]gluconolactone to monitor response to TMZ *in vivo*. Rats bearing orthotopic U251 tumor xenografts were treated with TMZ and both tumor volume and hyperpolarized δ-[1-^13^C]gluconolactone metabolism were measured longitudinally on days 0, 4 and 8 in order to determine whether hyperpolarized δ-[1-^13^C]gluconolactone can provide an early readout of treatment response. As shown in the representative T2-weighted MR images in **Figures 9A–C**, TMZ caused a reduction in tumor volume at day 8, but not at day 4. This temporal delay before MRI-detectable volumetric alterations caused by TMZ can be observed is highlighted in the quantification of tumor volume shown in **Supplementary Figure 11A**, indicating that significant tumor shrinkage can be observed only at later timepoints (> day 12). Importantly, metabolic heatmaps show a reduction in the tumor-localized ratio of [1-^13^C]6PG to δ-[1-^13^C]gluconolactone at a timepoint (day 4) when tumor volume is unchanged, an effect that is retained at day 8 (**Figures 9D–F**). Similar results were obtained when metabolic heatmaps of the ratio of [1-^13^C]6PG to total [1-^13^C]gluconolactone were examined (**Supplementary Figures 11B-D**). As shown in **Figure 9G**, quantification of the ratio of [1-^13^C]6PG to δ-[1-^13^C]gluconolactone or to total [1-^13^C]gluconolactone from an ROI within the tumor showed a significant reduction at day 4 relative to day 0 while there was no significant difference between day 4 and day 8. There was no significant difference in the ratio of [1-^13^C]6PG to δ-[1-^13^C]gluconolactone or to total [1-^13^C]gluconolactone between any of the timepoints when an ROI within the normal-appearing contralateral brain was analyzed (**Supplementary Figure 11E**). Collectively, the results in this section indicate that metabolic imaging of PGLS activity using hyperpolarized δ-[1-^13^C]gluconolactone has the ability to provide an early readout of response to TMZ that precedes the onset of MRI-detectable tumor shrinkage.

## Discussion

Tumors reprogram bioenergetics and redox to facilitate proliferation (7–9). Glucose metabolism through the PPP enables the synthesis of NADPH, which, in turn, facilitates GSH homoestasis (11, 12, 36). However, the role of PGLS in PPP-mediated redox in cancer remains unexplored. Here, we show that PGLS silencing in glioblastoma cells reduces steady-state levels of NADPH and GSH and increases levels of ROS, an effect that is linked to reduced clonogenicity. PGLS expression and activity as well as steady-state NADPH and GSH are higher in tumor tissues relative to normal-appearing contralateral brain tissue. Importantly, metabolic imaging of PGLS activity *in vivo* using hyperpolarized δ-[1-^13^C]gluconolactone can non-invasively monitor tumor burden and response to therapy in preclinical glioblastoma models.

The PPP is essential for most cells, including tumor cells, because the oxidative branch of the PPP produces reducing power in the form of NADPH that is needed for lipid biosynthesis and redox maintenance (12, 18, 28, 36). Several studies have probed the contributions of glucose-6-phosphate dehydrogenase and 6-phosphogluconate dehydrogenase to NADPH production and have identified these enzymes as therapeutic targets for cancer, including glioblastoma (13, 16, 19, 20). In contrast, although it was identified ~65 years ago (40), PGLS has been a much-neglected enzyme, partly because the substrate 6-phospho-δ-gluconolactone was thought to be unstable and to hydrolyze rapidly in a spontaneous manner (41). However, studies using ^31^P and ^13^C MRS analysis of the PPP indicate that 6-phospho-δ-gluconolactone, which is in equilibrium with 6-phospho-γ-gluconolactone, has a non-negligible lifetime, does not readily hydrolyze under physiological conditions and requires PGLS for conversion to 6PG in cells (14, 15, 42). Nevertheless, it is not clear if PGLS plays a role in cellular NADPH generation or redox homeostasis. Our findings indicate that silencing PGLS in multiple glioblastoma models (U87, U251 and GS2), including a clinically relevant patient-derived model (GS2), reduces steady-state levels of NADPH and GSH. Concomitantly, PGLS silencing increases ROS and reduces clonogenicity. Therefore, our study, to the best of our knowledge for the first time, identifies an essential role for PGLS in maintaining redox and relieving oxidative stress and points to PGLS as a potential therapeutic target in glioblastomas.

Our studies also indicate that PGLS expression and activity as well as NADPH and GSH are higher in glioblastoma tumor tissues relative to normal brain, while levels of ROS are lower. In this context, it should be noted that our results point to an inverse correlation between ROS levels and levels of GSH and NADPH, both when comparing tumor to normal brain, when comparing glioblastoma cells in which PGLS is silenced to their corresponding controls and when comparing TMZ-treated glioblastoma cells with vehicle-treated controls. These results are in line with previous studies that have identified negative correlations between GSH and markers of oxidative damage including ROS levels in mammalian cells (43–47). Although the relationship between GSH, NADPH and ROS is undeniably complex and governed by several factors such as subcellular compartmentalization, proliferative signaling and redox metabolism (47, 48), nevertheless, our results are consistent with prior studies that point to a key role for GSH and NADPH in ameliorating the deleterious effects of ROS (43–47).

At present, measurement of PPP flux and metabolic intermediates depends on kinetic analysis of ^13^C glucose-labeled cell or tissue extracts using liquid chromatography coupled with tandem mass spectrometry (11). While undoubtedly superior in sensitivity, mass spectrometry is inherently invasive and destructive. Studies have also used infusion of ^13^C glucose followed by thermally-polarized ^13^C MRS to measure PPP flux *in vivo* (49). Although this method is non-invasive, the lower sensitivity of thermally polarized ^13^C MRS necessitates long scan times to achieve adequate SNR, which hampers its clinical utility. Hyperpolarized ^13^C MRS provides an alternate, rapid approach to measure metabolic fluxes *in vivo* and, in the context of the PPP, hyperpolarized [U-^13^C, U-^2^H]glucose has been shown to be useful to monitor flux to 6PG *in vivo* (50–52). Our findings identify hyperpolarized δ-[1-^13^C]gluconolactone as a complementary non-invasive probe for imaging PGLS activity, and thereby flux through the PPP, *in vivo*.

To the best of our knowledge, this is the first demonstration of the use of hyperpolarized δ-[1-^13^C]gluconolactone for imaging *in vivo*. Hyperpolarized δ-[1-^13^C]gluconolactone has been previously used to monitor 6PG production *ex vivo* in isolated perfused liver (27), but its utility *in vivo* or in cancer has not been evaluated. In terms of translational potential, δ-[1-^13^C]gluconolactone fulfills the requirements for a successful hyperpolarized ^13^C agent such as T_1_, chemical shift separation, safety and BBB permeability (a necessity for studies in the brain) (23). It has a sufficiently long T_1_ (~32 s) at the clinically relevant field strength of 3 T, a value comparable to that of hyperpolarized [2-^13^C]pyruvate (~39 s), which has been successfully used to probe TCA cycle metabolism in the brain in human studies (25, 53). The chemical shift separation of 1.6 ppm between the gamma form of the substrate (γ-[1-^13^C]gluconolactone; 177 ppm) and the product ([1-^13^C]6PG; 178.6 ppm) is sufficient to allow visualization of product formation at the spectral resolution of our *in vivo* studies at 3 T. The chemical shift between the two forms of the substrate δ-[1-^13^C]gluconolactone (173.8 ppm) and γ-[1-^13^C]gluconolactone (177 ppm) was also sufficient to visualize and quantify the substrate *in vivo* at 3 T. Importantly, the ability to quantify [1-^13^C]6PG production in healthy tumor-free brain points to sufficient BBB permeability. With regard to safety, no adverse events were observed in our rats at the concentration of δ-[1-^13^C]gluconolactone (37.8 mM) used in our studies. Importantly, δ-gluconolactone is commonly used as a food additive at concentrations ranging from 5 - 50 mM (54), further allaying concerns of toxicity.

Previous studies indicate that δ-[1-^13^C]gluconolactone is transported across cell membranes *via* glucose transporters (27, 55). Since glucose transporters are expressed universally in mammalian cells because of the central role of glucose in cell metabolism (56), our results highlight the potential utility of hyperpolarized δ-[1-^13^C]gluconolactone as a metabolic imaging agent in human health and disease. Notably, although our study is focused on brain tumors, hyperpolarized δ-[1-^13^C]gluconolactone could potentially be useful in other tumors that have elevated PPP flux, including low-grade gliomas, pancreatic ductal adenocarcinomas, breast and lung cancers (12, 17). In this context, it should also be noted that PPP flux is also known to be altered in non-cancerous brain lesions such as Alzheimer’s and Parkinson’s disease (57, 58), suggesting that hyperpolarized δ-[1-^13^C]gluconolactone has potential as a non-invasive probe of alterations in the PPP in these neurological disorders.

A major challenge in glioma imaging at present is the inability of MRI to distinguish between tumor and regions of gliosis, edema or necrosis (59). MRI is also insufficient for accurate assessment of treatment response and the failure to distinguish true response to therapy from pseudoprogression is a significant hurdle in the clinic (59–62). Monitoring metabolic alterations associated with tumor bioenergetics and redox using hyperpolarized ^13^C MRS has been shown to be useful for non-invasive identification of tumor burden in several preclinical cancer models, including glioblastomas (63). In addition, alterations in tumor metabolism can precede volumetric alterations and, therefore, hyperpolarized ^13^C MRS has the potential to allow early assessment of response to therapy (23–25). Our results indicate that monitoring the spatial distribution of [1-^13^C]6PG production from hyperpolarized δ-[1-^13^C]gluconolactone serves to demarcate tumor from surrounding normal-appearing contralateral brain, thereby highlighting the ability of hyperpolarized δ-[1-^13^C]gluconolactone to provide a readout of tumor burden *in vivo*. Importantly, our studies indicate that reduced [1-^13^C]6PG production from hyperpolarized δ-[1-^13^C]gluconolactone can serve as a metabolic imaging biomarker of response to TMZ, which is part of standard of care for glioblastoma patients (4, 5). Notably, the TMZ-induced reduction in [1-^13^C]6PG production from hyperpolarized δ-[1-^13^C]gluconolactone was observed at early timepoints that preceded the onset of volumetric alterations as assessed by T2-weighted MRI, suggesting that hyperpolarized δ-[1-^13^C]gluconolactone has the potential to report on pseudoprogression *in vivo*.

In summary, our study identifies, for the first time, a role for PGLS in redox homeostasis and tumor proliferation in glioblastomas. Importantly, we show that metabolic imaging of PGLS activity using hyperpolarized δ-[1-^13^C]gluconolactone has the ability to non-invasively assess tumor burden and early response to therapy in preclinical glioblastoma models *in vivo*, findings with important implications for patients with glioblastomas and, potentially, other disease states.

## Data Availability Statement

The raw data supporting the conclusions of this article will be made available by the authors, without undue reservation.

## Ethics Statement

The animal study was reviewed and approved by UCSF Institutional Animal Care and Use Committee (IACUC Protocol No: AN170079).

## Author Contributions

GB designed and performed *in vivo* experiments and wrote and revised the manuscript. CT synthesized δ-[1-^13^C]gluconolactone and reviewed the manuscript. PC and PL contributed to the development of *in vivo* imaging acquisition methods. AMG assisted with the cell studies. CN and ES assisted with experiments and reviewed the manuscript. DW oversaw δ-[1-^13^C]gluconolactone synthesis. RF oversaw δ-[1-^13^C]gluconolactone synthesis and reviewed the manuscript. SR conceived of the study, reviewed the manuscript, and secured funding. PV conceived of the study, designed and performed cell studies, wrote and revised the manuscript and secured funding for the study. All authors contributed to the article and approved the submitted version.

## Conflict of Interest

The authors declare that the research was conducted in the absence of any commercial or financial relationships that could be construed as a potential conflict of interest.

## Abbreviations

6PG, 6-phospho-gluconate; PGLS, 6-Phosphogluconolactonase; BBB, blood brain barrier; dDNP, dissolution dynamic nuclear polarization; DMEM, Dulbecco’s modified Eagle’s medium; DMSO, dimethyl sulfoxide; EPSI, echo planar spectroscopic imaging; GSH, glutathione; MRI, magnetic resonance imaging; MRS, magnetic resonance spectroscopy; PPP, pentose phosphate pathway; ROI, region of interest; ROS, reactive oxygen species; SNR, signal to noise ratio; TMZ, temozolomide.
