# Supplementary material for: Imaging 6-Phosphogluconolactonase Activity in Brain Tumors In Vivo Using Hyperpolarized δ-[1-13C]gluconolactone
Source: Front Oncol. 2021 Apr 15;11:589570. doi: 10.3389/fonc.2021.589570 (PMC8082394; doi:10.3389/fonc.2021.589570)
Supplement: Supplementary file 1 [file Image_1.pdf]

Supplementary Material

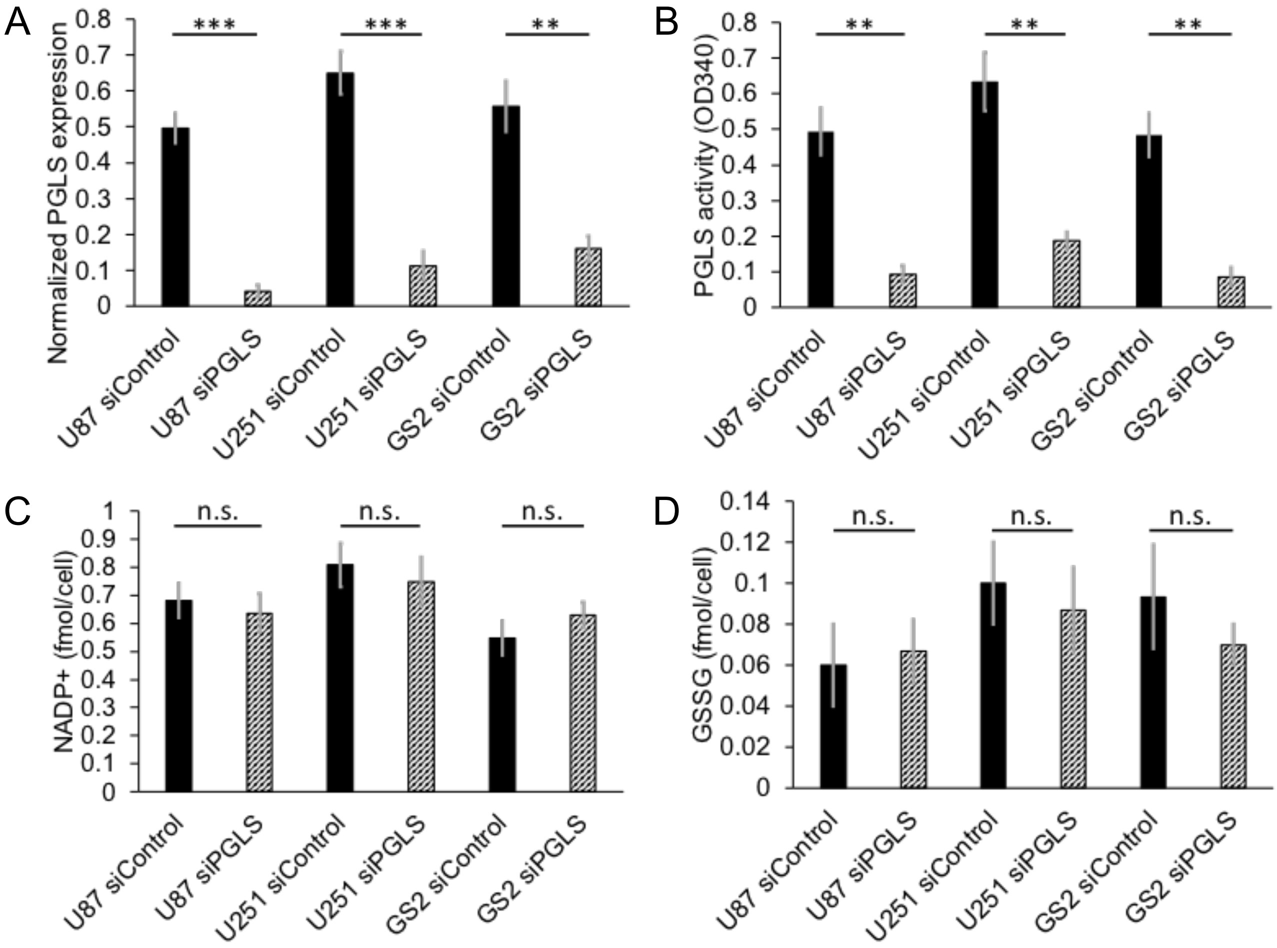

Supplementary Figure 1. Verification of PGLS silencing in glioblastoma models. Assessment of PGLS expression by quantitative RT-PCR **(A)** and PGLS activity **(B)** in U87, U251 and GS2 cells transfected with siRNA targeting PGLS (siPGLS; n = 3 each) or with non-targeting control siRNA (siControl; n = 3 each). Quantification of $NADP^+$ **(C)** and GSSG **(D)** levels in U87, U251 and GS2 siPGLS and siControl cells (n = 3 each). ** indicates p < 0.01, *** indicates p < 0.001.

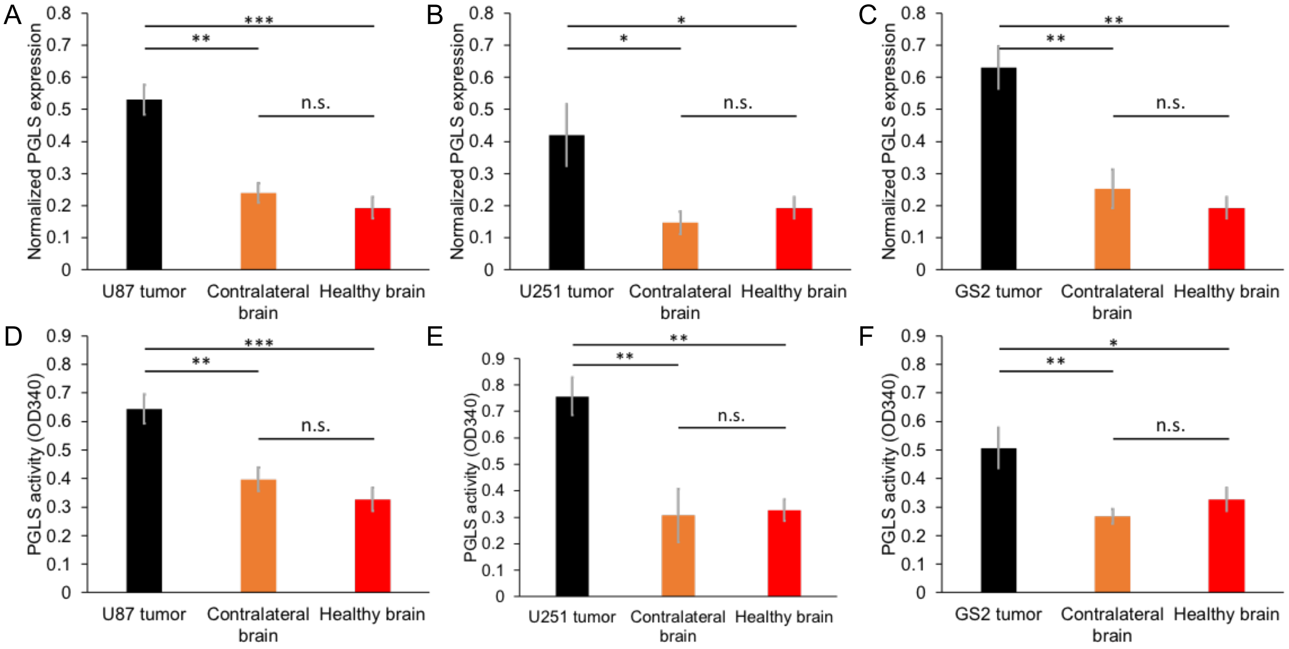

Supplementary Figure 2. PGLS expression and activity are higher in glioblastoma tumor tissues relative to normal brain. Quantification of PGLS mRNA by quantitative RT-PCR **(A-C)** and PGLS activity **(D-F)** in tumor tissues (black bars) and normal-appearing contralateral brain (orange bars) from rats bearing orthotopic U87, U251 or GS2 tumors or in healthy brain tissues (red bars) from tumor-free rats (n = 3 each). * indicates $p < 0.05$, ** indicates $p < 0.01$, *** indicates $p < 0.001$.

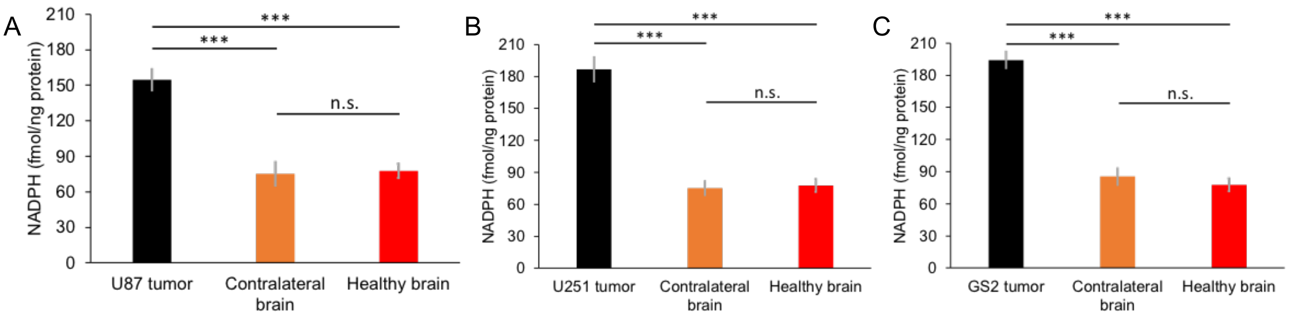

Supplementary Figure 3. Steady-state levels of NADPH are higher in glioblastoma tumor tissues relative to normal brain. Quantification of NADPH by spectrophotometric assay in tumor tissues (black bars) and normal-appearing contralateral brain (orange bars) from rats bearing orthotopic U87 **(A)**, U251 **(B)** or GS2 **(C)** tumors. Healthy brain tissues (red bars) from tumor-free rats were also examined. All experiments were done in triplicate (n = 3). *** indicates $p < 0.001$.

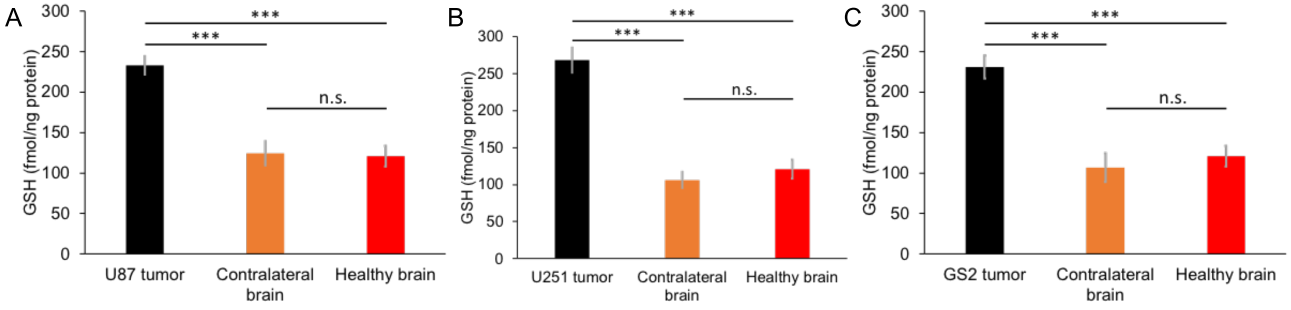

Supplementary Figure 4. GSH levels are higher in glioblastoma tumor tissues relative to normal brain. Quantification of GSH by spectrophotometric assay in tumor tissues (black bars) and normal-appearing contralateral brain (orange bars) from rats bearing orthotopic U87 **(A)**, U251 **(B)** or GS2 **(C)** tumors. Healthy brain tissues (red bars) from tumor-free rats were also examined. All experiments were done in triplicate (n = 3). *** indicates $p < 0.001$.

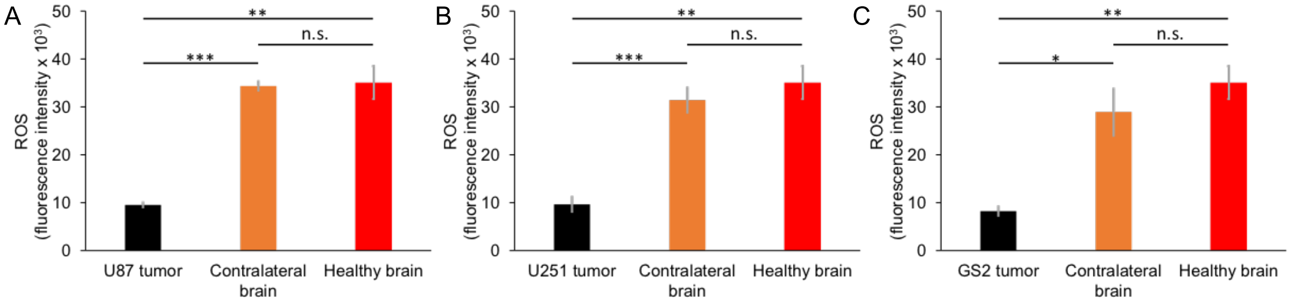

Supplementary Figure 5. Oxidative stress is lower in glioblastoma tumor tissues relative to normal brain. Quantification of ROS levels by fluorimetric assay in tumor tissues (black bars) and normal-appearing contralateral brain (orange bars) from rats bearing orthotopic U87 **(A)**, U251 **(B)** or GS2 **(C)** tumors. Healthy brain tissues (red bars) from tumor-free rats were also examined. All experiments were done in triplicate (n = 3). * indicates $p < 0.05$, ** indicates $p < 0.01$, *** indicates $p < 0.001$.

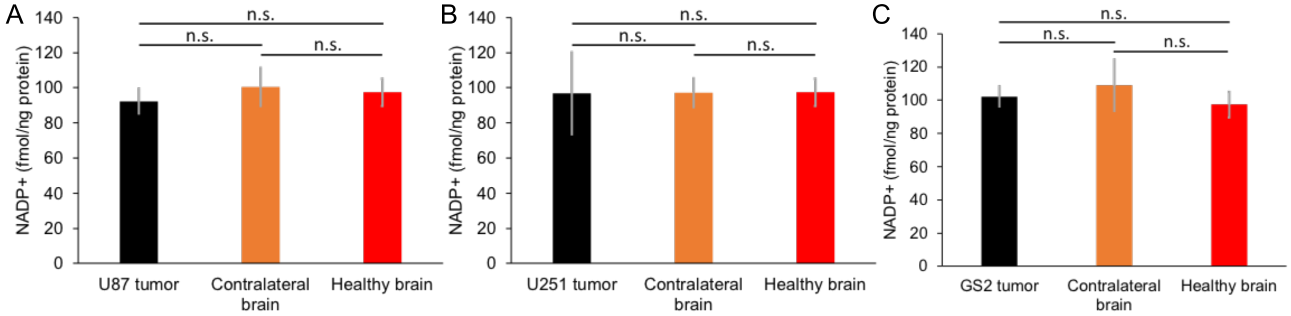

Supplementary Figure 6. $NADP^+$ levels do not differ between glioblastoma and normal brain tissues. Quantification of steady-state $NADP^+$ levels by spectrophotometric assay in tumor tissues (black bars) and normal-appearing contralateral brain (orange bars) from rats bearing orthotopic U87 **(A)**, U251 **(B)** or GS2 **(C)** tumors. Healthy brain tissues (red bars) from tumor-free rats were also examined. All experiments were done in triplicate (n = 3).

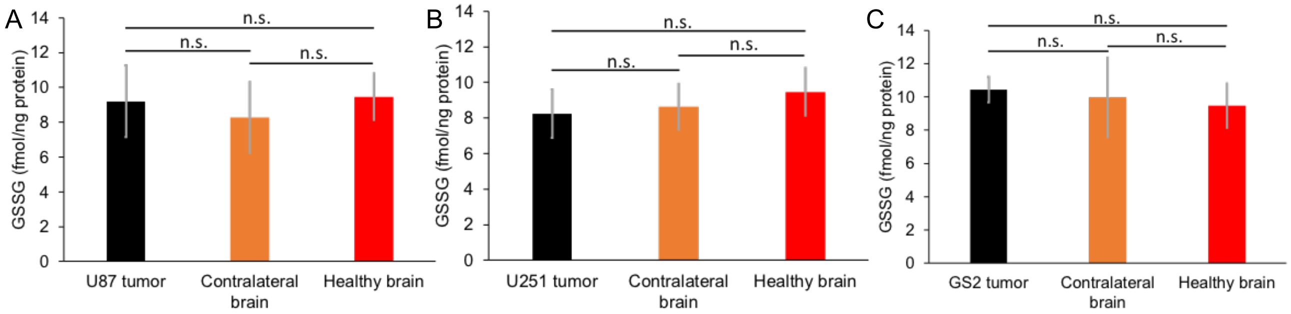

Supplementary Figure 7. GSSG levels do not differ between glioblastoma and normal brain tissues. Quantification of steady-state GSSG by spectrophotometric assay in tumor tissues (black bars) and normal-appearing contralateral brain (orange bars) from rats bearing orthotopic U87 **(A)**, U251 **(B)** or GS2 **(C)** tumors. Healthy brain tissues (red bars) from tumor-free rats were also examined. All experiments were done in triplicate (n = 3).

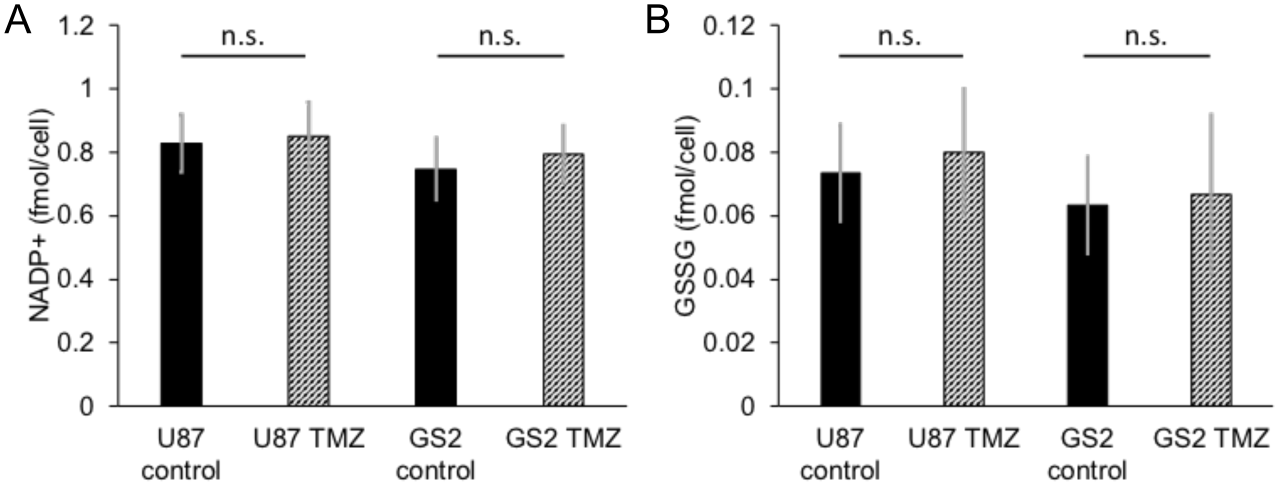

Supplementary Figure 8. TMZ does not affect $NADP^+$ or GSSG levels in glioblastoma cells. Effect of TMZ on steady-state $NADP^+$ **(A)** and GSSG (**B**) as measured by spectrophotometric assays in U87 and GS2 models.

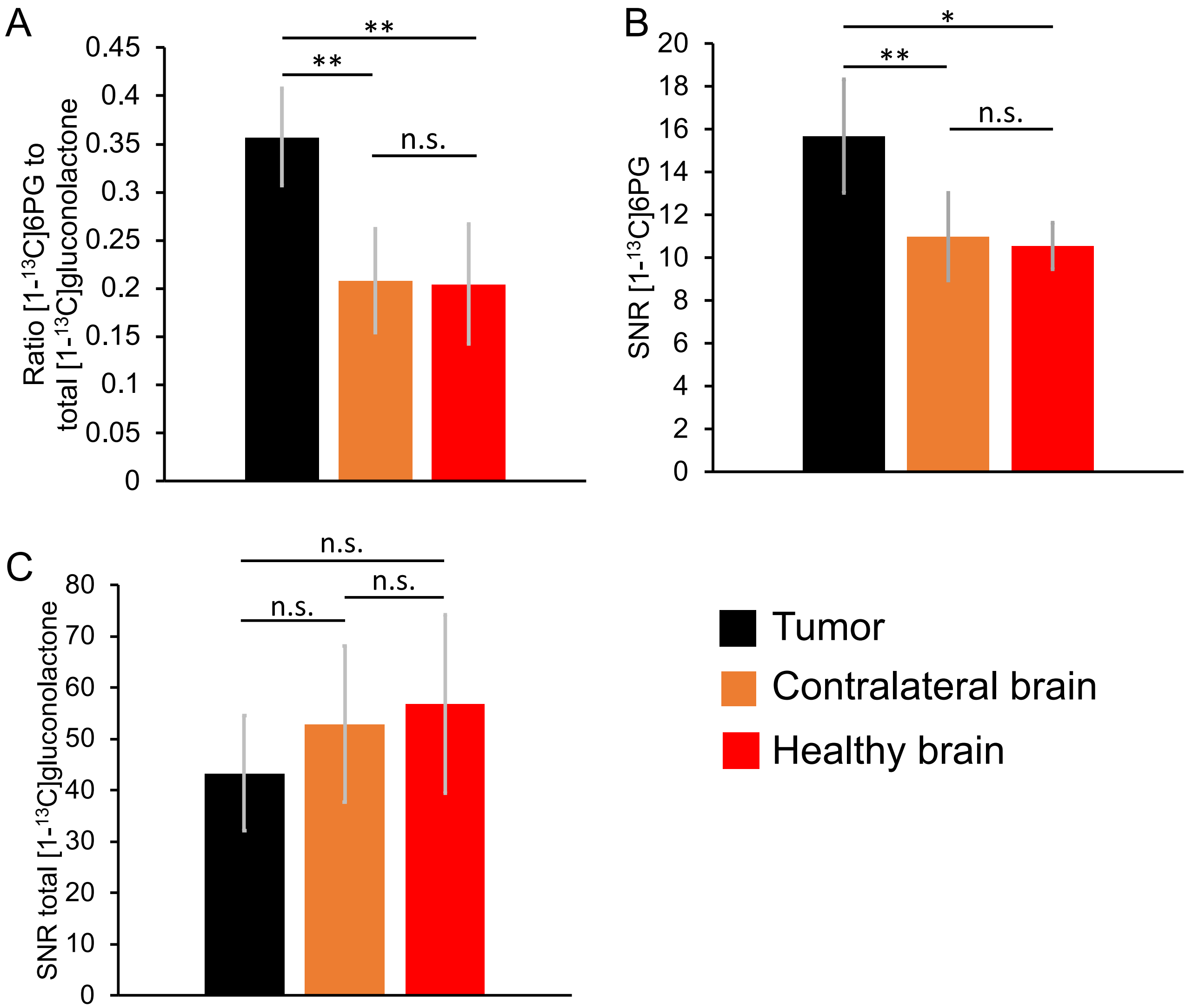

Supplementary Figure 9. Hyperpolarized δ-[1-^{13}C]gluconolactone metabolism to [1-^{13}C]6PG can differentiate tumor from normal brain *in vivo*. Quantification of the ratio of [1-^{13}C]6PG to total [1-^{13}C]gluconolactone **(A)**, the SNR of [1-^{13}C]6PG **(B)** and the SNR of total [1-^{13}C]gluconolactone **(C)** in ROIs from tumor (black), normal-appearing contralateral brain (orange) and healthy tumor-free brain (red) in the U87 model. n = 6 each; * indicates $p < 0.05$, ** indicates $p < 0.01$.

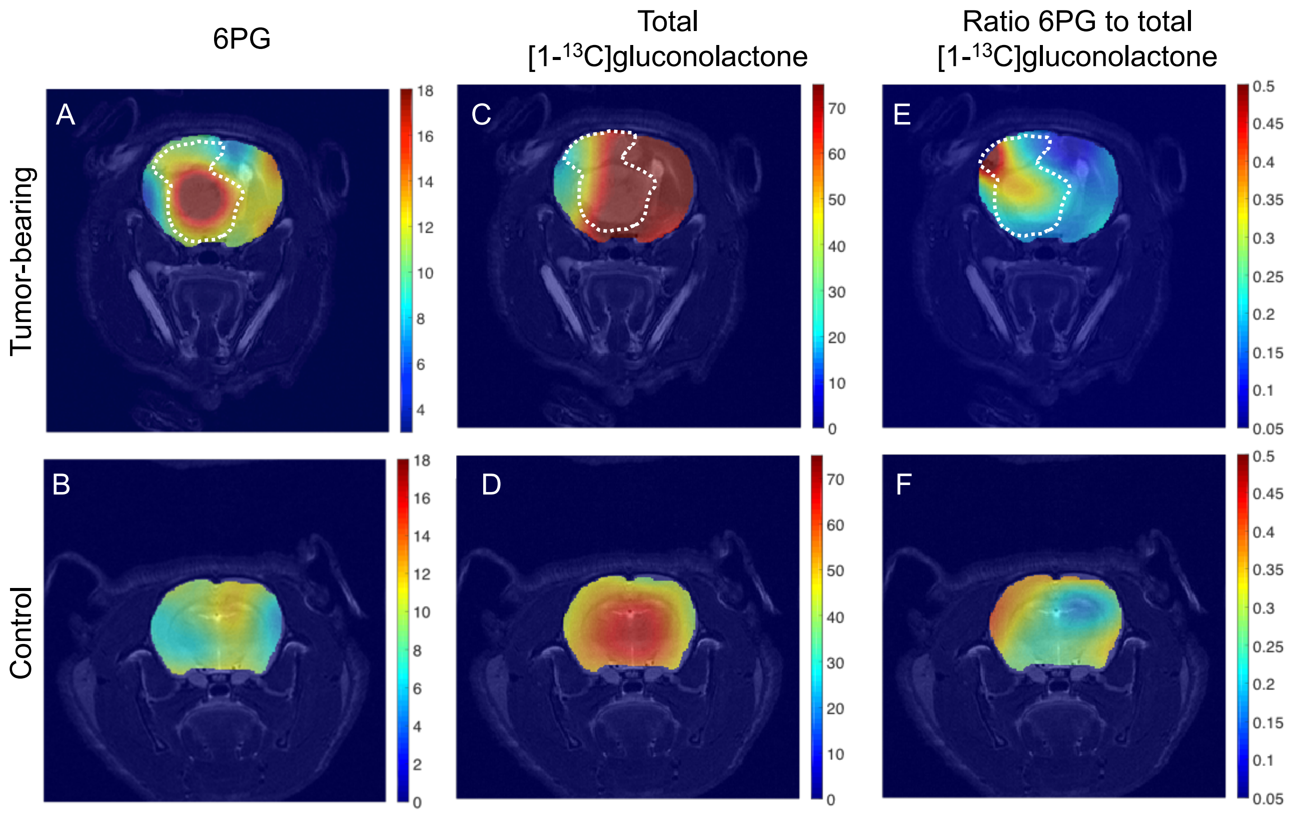

Supplementary Figure 10. Metabolic imaging of glioblastoma *in vivo* using hyperpolarized δ-[1-^{13}C]gluconolactone. Representative metabolic heatmaps of the SNR of [1-^{13}C]6PG **(A-B)**, the SNR of total [1-^{13}C]gluconolactone **(C-D)** the ratio of [1-^{13}C]6PG to total [1-^{13}C]gluconolactone **(E-F)** from a rat bearing an orthotopic U87 tumor or from tumor free healthy control. The tumor region is contoured by white dotted lines.

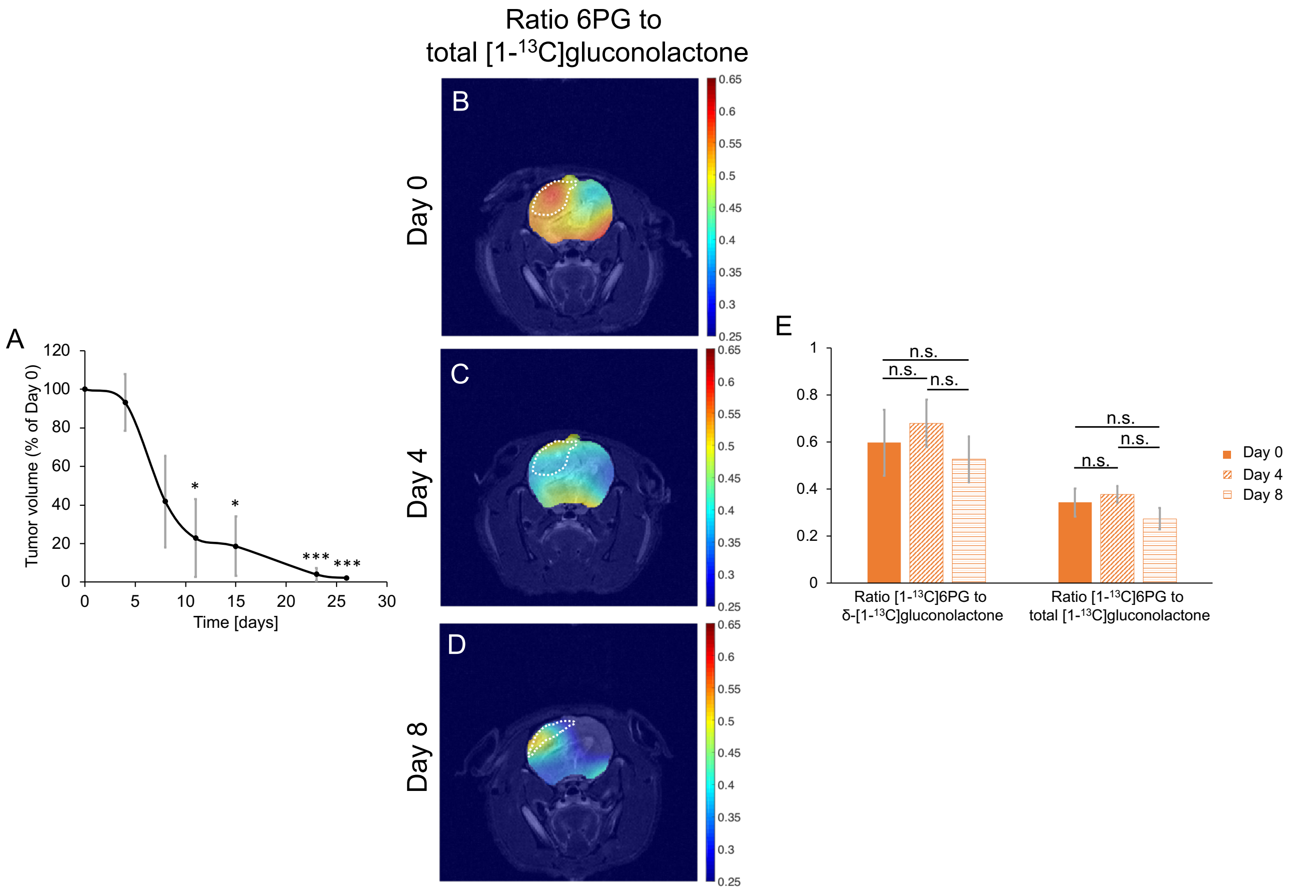

Supplementary Figure 11. Hyperpolarized δ-[1-^{13}C]gluconolactone can be used to monitor response to TMZ *in vivo*. (A) Longitudinal analysis of tumor volume as measured by T2-weighted MRI in rats bearing orthotopic U251 tumor xenografts treated with TMZ. Representative metabolic heatmaps of the ratio of [1-^{13}C]6PG to total [1-^{13}C]gluconolactone **(B-D)** from a rat bearing an orthotopic U251 tumor xenograft at day 0, day 4 and day 8 following treatment with TMZ. The tumor region is contoured by white dotted lines. **(E)** Quantification of the ratio of [1-^{13}C]6PG to δ-[1-^{13}C]gluconolactone or the ratio of [1-^{13}C]6PG to total [1-^{13}C]gluconolactone from an ROI within the normal-appearing contralateral brain at day 0, day 4 and day 8 of TMZ treatment in rats bearing orthotopic U251 tumor xenografts (n = 4 at day 0 and n = 3 at day 4 and 8). * indicates $p < 0.05$, *** indicates $p < 0.001$.
